# Risk of Stroke in Patients with Stable Coronary Artery Disease Undergoing Percutaneous Coronary Intervention versus Optimal Medical Therapy: Systematic Review and Meta-Analysis of Randomized Controlled Trials

**DOI:** 10.1371/journal.pone.0158769

**Published:** 2016-07-08

**Authors:** Nevio Taglieri, Maria Letizia Bacchi Reggiani, Gabriele Ghetti, Francesco Saia, Gianni Dall’Ara, Pamela Gallo, Carolina Moretti, Tullio Palmerini, Cinzia Marrozzini, Antonio Marzocchi, Claudio Rapezzi

**Affiliations:** Istituto di Cardiologia, Dipartimento di Medicina Specialistica, Diagnostica e Sperimentale, Alma Mater Studiorum Università di Bologna, Bologna, Italy; University of Messina, ITALY

## Abstract

**Background:**

Stroke is a rare but serious adverse event associated with percutaneous coronary intervention (PCI). However, the relative risk of stroke between stable patients undergoing a direct PCI strategy and those undergoing an initial optimal medical therapy (OMT) strategy has not been established yet. This study sought to investigate if, in patients with stable coronary artery disease (SCAD), an initial strategy PCI is associated with a higher risk of stroke than a strategy based on OMT alone.

**Methods:**

We performed a meta-analysis of 6 contemporary randomized control trials in which 5673 patients with SCAD were randomized to initial PCI or OMT. Only trials with stent utilization more than 50% were included. Study endpoint was the rate of stroke during follow up.

**Results:**

Mean age of patients ranged from 60 to 65 years and stent utilization ranged from 72% to 100%. Rate of stroke was 2.0% at a weighted mean follow up of 55.3 months. On pooled analysis, the risk of stroke was similar between patients undergoing a PCI plus OMT and those receiving only OMT (2.2% vs. 1.8%, OR on fixed effect = 1.24 95%CI: 0.85–1.79). There was no heterogeneity among the studies (I^2^ = 0.0%, P = 0.15). On sensitivity analysis after removing each individual study the pooled effect estimate remains unchanged.

**Conclusions:**

In patients with SCAD an initial strategy based on a direct PCI is not associated with an increased risk of stroke during long-term follow up compared to an initial strategy based on OMT alone.

## Introduction

In patients with stable coronary artery disease (SCAD) the role of percutaneous coronary intervention (PCI) is controversial. Indeed, data from recent randomized clinical trials (RCT) [1–3] and meta-analyses[4–6] have consistently shown that in patients with SCAD, PCI does not reduce the risk of death and myocardial infarction (MI) compared with optimal medical treatment (OMT). Furthermore the attempts to identify patients at higher risk, such as those with documented myocardial ischemia[7] or diabetes[2] that would benefit from an interventional strategy have failed. On the other hand, in most of the randomized comparisons, the patients with SCAD treated with PCI had a better and faster symptoms relief than those treated medically. Yet, a substantial proportion of patients belonging to the latter group crossed over and underwent PCI. Thus, the choice of referring patients to an initial conservative strategy based on either the optimization of medical treatment or a direct PCI strategy plus OMT should take into account several factors such as the presence and the intensity of symptoms, the patient’s risk profile (for example patients with marked signs of myocardial ischemia, systolic left ventricle dysfunction, high risk coronary obstructions, comorbidities that have been scarcely represented in randomized clinical trials) and intervention hazards. Regarding this latter aspect, stroke following PCI represents undoubtedly a dismal complication with severe disability and high mortality[8,9]. Data from large registries [8,9] suggest that guiding catheter manipulation, use of anticoagulant and potent antiplatelet inhibitors (such as glycoprotein IIb/IIIa), especially in technically difficult PCI [9], may increase the risk of ischemic and hemorrhagic stroke, respectively. However, the relative risk of stroke between stable patients undergoing a direct PCI strategy and those undergoing an initially conservative strategy has not been established yet. Indeed, all RCTs comparing PCI with OMT in stable patients were far underpowered to detect a difference in terms of such a rare event as stroke. Therefore, we performed a systematic review and meta-analysis of contemporary RCTs to investigate if PCI plus OMT is associated with a higher risk of stroke than OMT alone in patients with SCAD.

## Methods

### Study selection and study endpoint

We carried out a systematic review of the available publications according to the current PRISMA guidelines to perform meta-analyses of RCT[10] (S1 Table). We searched for relevant articles, filtered by RCT, published in the MEDLINE and the Cochrane Library using the following key words that were variously combined (S1 Text): “stable angina”, “stable coronary artery disease”, “medical therapy”, “PCI”, “stent”. No language restriction was used. We also checked the reference lists of reviews and relevant articles.

Inclusion criteria were as follows. *1*. RCT design, *2*. Comparison between PCI plus OMT and OMT alone, *3*. Stent implantation in more than 50% of PCI patients *4*. Data on stroke available.

For studies that compared OMT with separate arms of PCI and coronary bypass only data on comparison between OMT and PCI were extracted.

We excluded trials that included patients with a recent (within 6 months) episode of acute coronary syndrome. Trials that compared OMT with revascularization as a whole (either PCI or coronary bypass) were also excluded. Two investigators (N.T, G.G.) independently reviewed the titles, abstracts, and studies to determine whether they met the inclusion criteria. Conflicts between reviewers were resolved by consensus.

The study endpoint was the rate of stroke based on the definition applied in each study. For the endpoint we used data from the longest follow up available to a maximum of 5-year follow up.

Data were extracted on the basis of the intention-to-treat populations.

### Statistical Analysis

Odd Ratios (OR) and 95% Confidence Intervals (95% CI) were used as the summary statistic. The pooled OR was calculated by using both fixed-effect (inverse variance weighted) and random-effect (DerSimonian and Laird) models. Between-study heterogeneity of effects was analyzed using the χ^2^ and inconsistency across study results quantified by I^2^ statistics, with I^2^ <25%, 25% ≤ I^2^ ≤50%, and I^2^ > 50%, respectively, representing mild, moderate, and severe inconsistency.

Sensitivity analysis was performed by evaluating the influence of removing individual studies on the pooled OR. The possibility of publication bias was assessed both visually by funnel plot and Harbord’s test. Statistical analyses were performed using Stata/SE 11.2 (StataCorp LP, College Station, TX).

## Results

**Fig 1:**
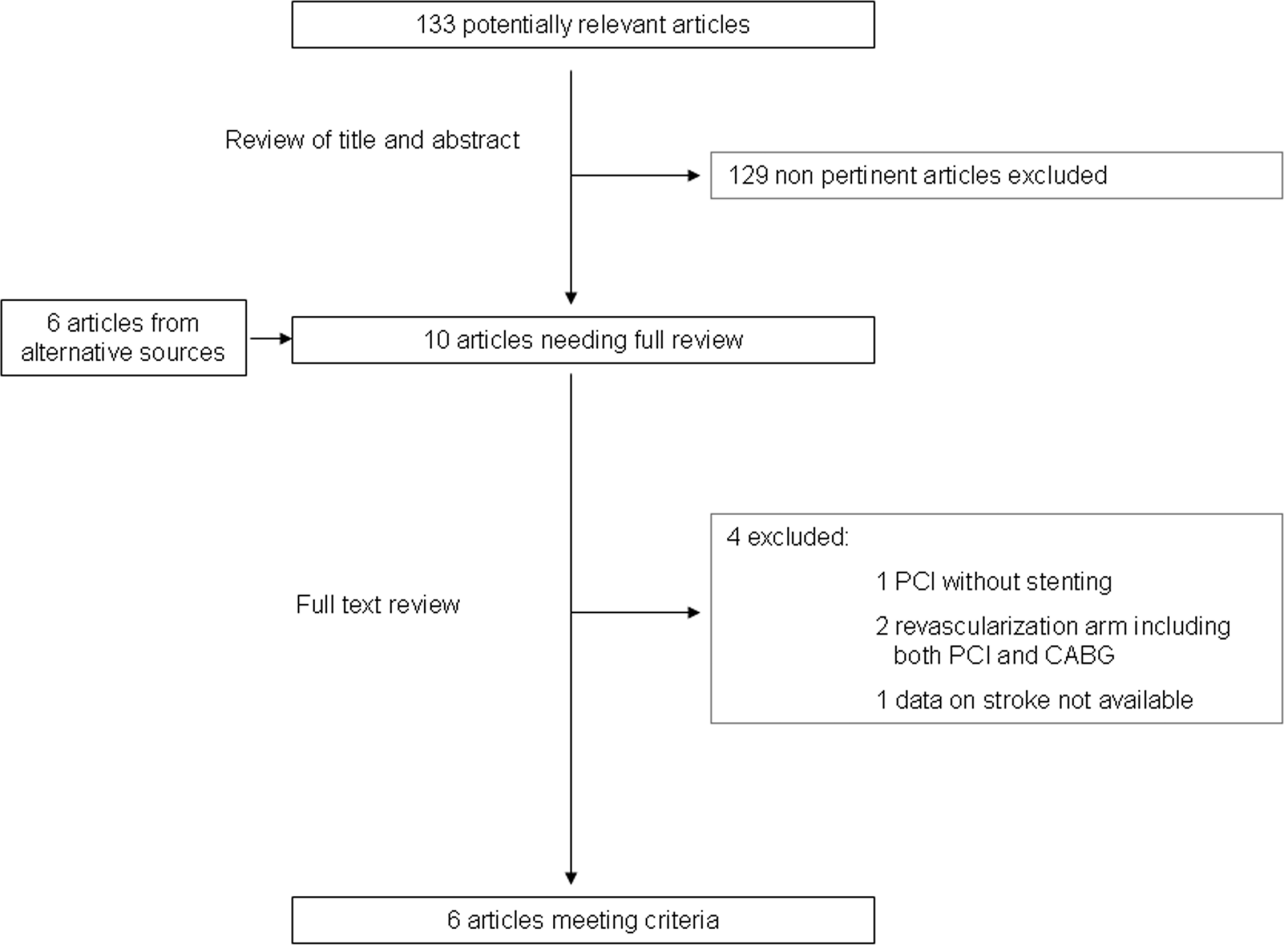
Study flow chart. Abbreviations: CABG = coronary artery bypass grafting; PCI = percutaneous coronary intervention.

Fig 1 shows the flow chart for the study analysis (Poster presentation at TCT 2015; http://content.onlinejacc.org/article.aspx?articleid=2452937). Of 133 potentially relevant articles initially screened, 6 met the inclusion criteria and were included in the meta-analysis with a total of 5673 patients[1–3,11–13] (S2 Text). Table 1 shows the main characteristics of the studies included. Stent utilization ranged from 72% to 100%. Definition of optimal medical therapy was provided in all studies but one. Most of the studies had 5-year follow up available[1,2,13,14], 2 studies reported data on a shorter follow up (1-year[12] and 2-year[3], respectively).

**Table 1:** Characteristics of Included Trials.

Trial name or first Author	Yearsof enrollment	Year of publication	Design of the trial	Inclusion criteria	Exclusion criteria	Primary endpoint	Number of patients	Definition of OMT	Stroke Definition	Stent use (%)	Follow up (months)
**MASS II[11,14]**	1997–2001	2004	Single-center RCT	Angiographically documented proximal multivessel disease (>70% stenosis) **and** documented ischemia (positive stress testing or stable angina CCS II-III)	Unstable angina,acute MI, LVEF < 40%, previous PCI or CABG, single vessel disease, LMA stenosi ≥ 50%	composite of: cardiac death- MI- refractory angina	408	Nitrates, Aspirin, Beta-blockers, CCB, ACE-I,Statin	onset of neurological deficit associated with structural compatible lesion identified by CT scan or MRI”	72	60
**Hambrecht et al.[12]**	1997–2001	2004	Single-center RCT	Male < 70 yr,Stable-angina (CCS I-III) **and** documented myocardial ischemia by stress-test **and** at least 1vessel disease (≥ 75% by visual assessment)	ACS < 2 months, LMA > 25%, high-grade LAD proximal disease, LVFE < 40%, Insulin dependent diabetes mellitus, CABG or PCI within previous 12 months	ischemic events:- cardiac death- stroke- resuscitation after cardiac arrest- CABG- angioplasty- worsening angina requiring hospitalization	101	exercise training **AND** guidelines recommended medical therap	sudden focal disturbance of brain function of presumed vascular origin persisting longer than 24 hours	100	12
**COURAGE[1]**	1999–2004	2007	Multicenter RCT	Stable CAD **or** medically stabilized CCS IV angina **plus** stenosis ≥70% in at least 1 of proximal epicardial coronary artery **and** objective of myocardial ischemia **or** stenosis >80% **and** typical angina without provocative tests.	CCS IV angina, markedly positive stress test, refractory heart failure,LVEF < 30%, revascularization within the previous 6 months	composite of:- death- non fatal MI	2287	aspirin (or clopidogrel) long acting metoprolol, amplodipine, isosorbide mononitrate, lisiniopril(or losartan) simvastatin (alone or in combination with ezetimibe)	Not reported	94	60
**JSAP[13]**	1991–1997	2003	Single-center RCT	30–75 yr Stable low-risk CAD **and** 1 or 2 vessel disease (≥ 75% according to AHA classification or ≥ 60% on quantitative CA)	high-risk CAD (3 vessel, left main or ostial LAD); chronic total obstruction; ACS; LVEF < 50%; PCI not indicated; tendency to bleed or severe pneumonia; previous CABG with graft stenosis as responsible lesion; PCI or OMT already prescribed	Composite of:- death- ACS- cerebrovascular accidents- emergency hospitalization	384	NA	Sudden focal disturbance in brain function of presumed vascular origin persisting for longer 24 h	75	60
**BARI 2D[2]**	2001–2005	2009	Multicenter RCT (2 x 2 factorial design)	Type 2 diabetes mellitus **plus** CAD (≥50% stenosis of major epicardial coronary artery and a positive stress test**or** ≥70% stenosis associated with typical angina)	Required immediate revascularization, LMA disease, creatinine > 2.0 mg/dl,lycated Hb > 13%, NYHA Class III or IV, PCI or CABG within the previous 12 months, liver dysfunction	Overall mortality	1605	as guideline recommendation	rapid onset of persistent neurologic deficit attributed to an obstruction or ropture of the brain arterial system. The deficit is not known to a be secondary to brain trauma, tumor, infarction or other cause. The deficit must last more than 24 hours unless death supervenes or there is demonstrable lesion on CT or MRI compatible with an acute stroke	91	60
**FAME 2[3]**	2010–2012	2012	Multicenter RCT	Stable CAD **or**angina pectoris CCS class 4 subsequently stabilized medically (minimum 7 days); **or** atypical or no chest pain but documented ischemia on non-invasive testing; **plus**At least one stenosis ≥50% in at least one major native coronary artery (≥ 2.5mm) and supplying viable myocardium **and**eligible for PCI **and** FFR value <80%	Age < 21 y;CABG best treatment; LMA disease requiring revascularization;Less than 1 week STEMI or Non-STEMI; Prior CABG; Contra-indication to dual antiplatelet therapy; LVEF < 30%;Severe LV hypertrophy (defined as a septal wall thickness at echocardiography ofmore than 13 mm);concomitant need for valvular or aortic surgery; Extremely tortuous or calcified coronary arteries precluding FFR measurements;Life expectancy < 2 years;	composite of:- death- non fatal MI- urgent revascularization	888	aspirin, beta-blocker,ACE-I (or ARB),Atorvastatin (alone or in combination with ezetimibe)	Not reported	100 (II-generationDES)	24

Abbreviations: ACE-I = angiotensin converting enzyme inhibitors; ACS = acute coronary syndrome; AHA = American Heart Association; ARB = angiotensin receptor blockers; BARI 2 = Bypass Angioplasty Revascularization Investigation 2 Diabetes; CA = coronary angiography; CABG = coronary artery bypass grafting; CAD = coronary artery disease; CCB = calcium channel blockers; CCS = Canadian cardiac society; COURAGE = Clinical Outcome Utilizing Revascularization and Aggressive Drug Evaluation; CT = Computer Tomography, DES = drug eluting stent, FFR = fractional flow reserve; FAME 2 = Fracional Flow Reserve versus Angiography for Multivessel Evaluation 2; JSAP = Japanese stable angina pectoris; LAD = left anterior descendent artery;; LMA = left main artery; LVEF = left ventricular ejection fraction; MASS II = The Medicine, Angioplasty, or Surgery Study II; MRI = magnetic resonance imaging, NYHA = New York Heart Association; OMT = optical medical therapy; RCT = randomized controlled trial; MI = myocardial infarction; PCI = percutaneous coronary intervention; STEMI = ST elevation myocardial infarction.

Mean age of patients ranged from 60 to 65 years with a weighted mean age of 62.3 years. All studies enrolled predominantly male patients (Table 2). Utilization of aspirin was equal or greater than 80% in all studies. Beta-blockers and statins were used in more than 60% of patients with the exception of 1 trial[13] in which they were used in nearly 50%. Angiotensin converting enzyme inhibitors or angiotensin receptor blockade were used in more than 60% in most studies.

**Table 2:** Characteristics of patients.

	MASS II^11^		Hambrecht et al^12^		COURAGE^1^		JSAP^13^		BARI 2D^2^		FAME 2^3^	
	OMT + PCI	OMT	OMT + PCI	OMT	OMT + PCI	OMT	OMT + PCI	OMT	OMT + PCI	OMT	OMT + PCI	OMT
Characteristic												
Patients (n)	205	203	50	51	1149	1138	188	191	798	807	447	441
Age, mean (yr)	60	60	60	62	62	62	65	64	62	62	64	64
Male (%)	67	69	100	100	85	85	75	75	68	70	80	77
Prior MI (%)	52	39	39	52	38	39	14	15	30	32	37	38
Diabetes (%)	23	36	22	23	32	35	40	40	100	100	28	27
Multivessel disease (%)	100	100	40	54	69	70	33	33	20	30	44	41
Medical therapy (%)												
Aspirin	80	80	98	98	96	95	92	91	94	94	87	90
Beta blocker	61	68	86	88	85	89	44	52	84	88	76	78
ACE-I or ARB	30	29	88	74	62	65	42	39	91	92	69	70
Statin	73	68	80	72	86	89	49	45	95	95	83	82

ACE-I = angiotensin converting enzyme inhibitors; ARB = angiotensin receptor blockers; BARI 2 = Bypass Angioplasty Revascularization Investigation 2 Diabetes; COURAGE = Clinical Outcome Utilizing Revascularization and Aggressive Drug Evaluation; FAME 2 = Fracional Flow Reserve versus Angiography for Multivessel Evaluation 2; JSAP = Japanese stable angina pectoris study; MASS II = The Medicine, Angioplasty, or Surgery Study II, MI = myocardial infarction, OMT = optimal medical therapy; PCI = percutaneous coronary intervention.

**Fig 2:**
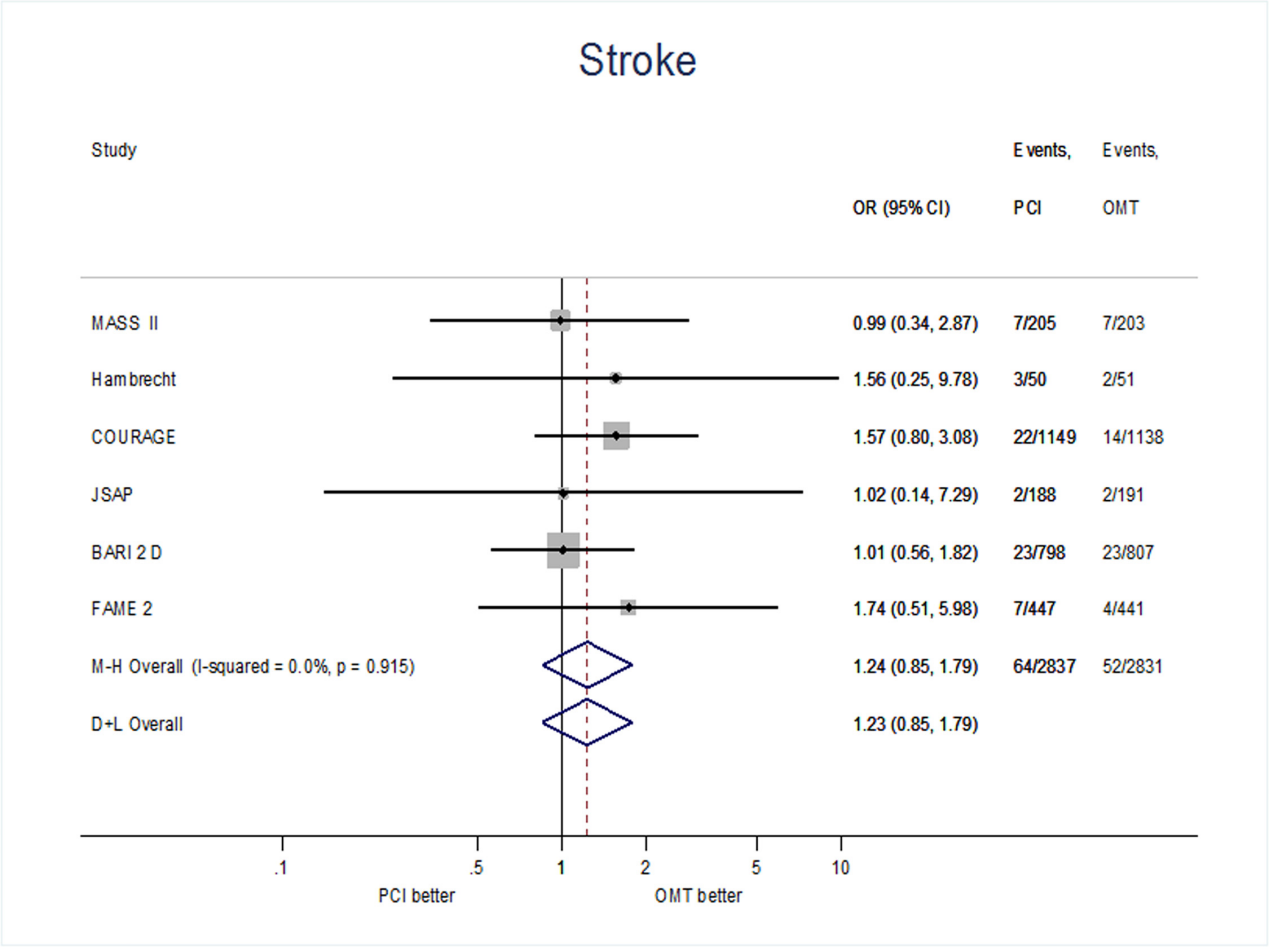
Risk of stroke in patients with PCI based strategy vs. patients with OMT based strategy. Abbreviations: CI = confidence intervals.

Among the 5668 patients included in the main analysis [in the Japanese stable angina pectoris study (JSAP) [13] outcomes were not reported for 5 patients) the rate of stroke was 2.0% at a weighted mean follow up of 55.3 months. On pooled analysis, the risk of stroke was similar between patients undergoing PCI plus OMT and those receiving only OMT (2.2% vs. 1.8%, OR on fixed effect = 1.24 95%CI: 0.85–1.79). There was no heterogeneity among the studies (I^2^ = 0.0%, P = 0.915) [Fig 2].

**Fig 3:**
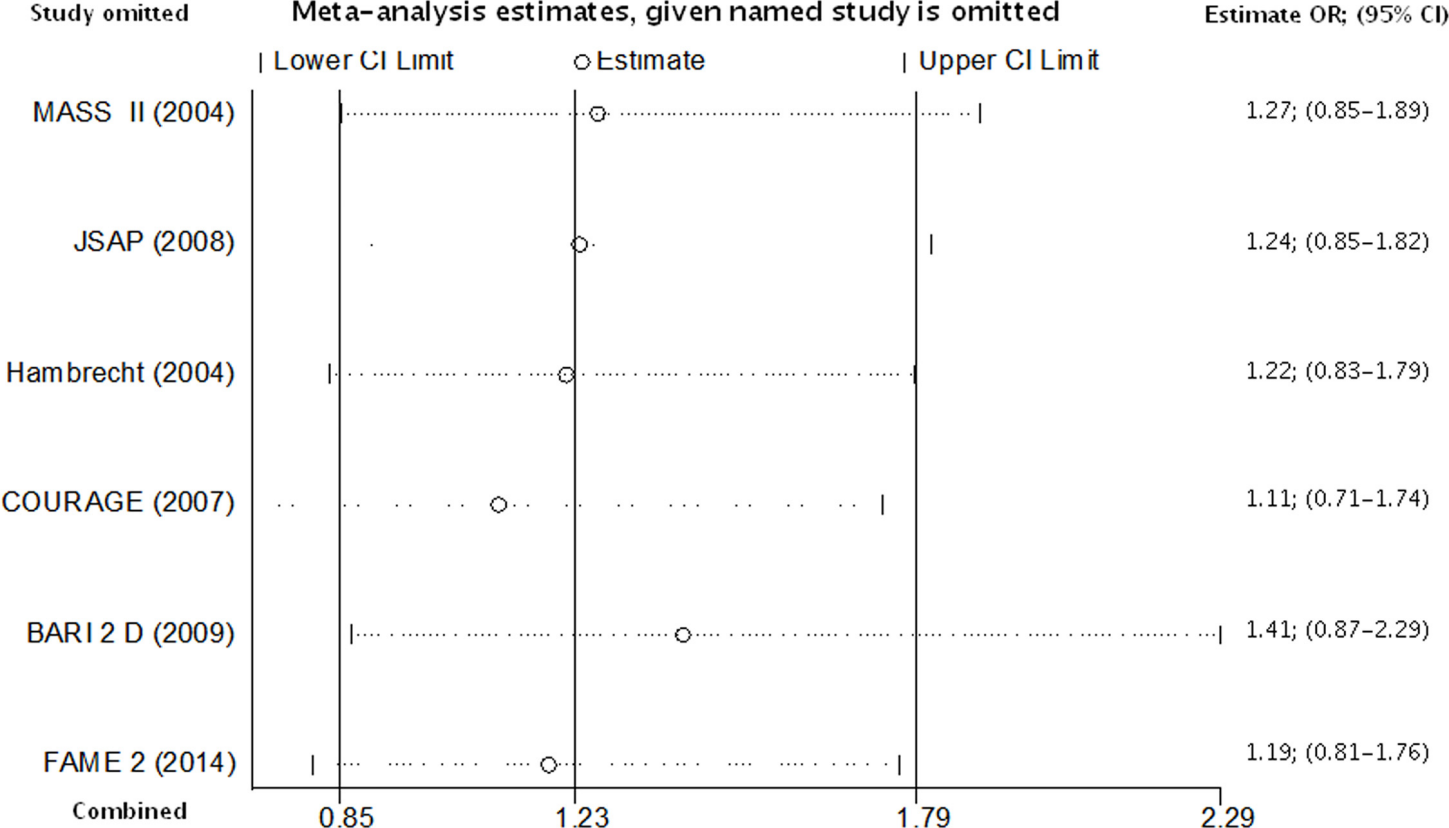
Risk of stroke in patients with PCI based strategy vs. patients with OMT based strategy. OR estimates given named study is omitted. Abbreviations: CI = confidence intervals OR = odds ratio.

Fig 3 shows that after removing each individual study the pooled effect estimate remains unchanged.

**Fig 4:**
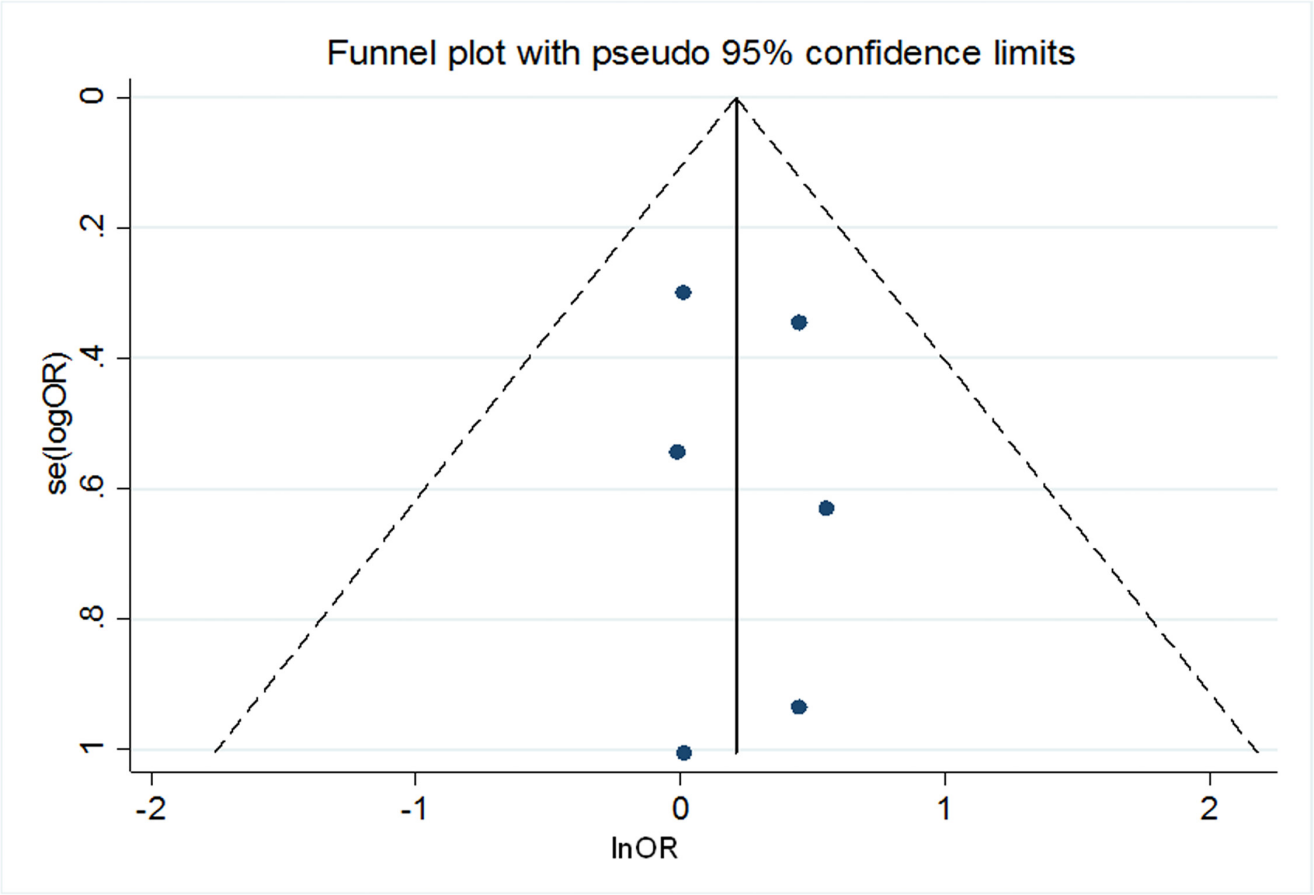
Estimation of publication bias. A funnel plot of SE versus ln(OR) for the meta-analysis. When the standard error is used on the *y* axis of a funnel plot, it is conventional to reverse the axis so that the most precise studies are displayed at the top of the plot. They tend to cluster near the mean effect size. Smaller studies are depicted at the bottom of the graph and tend to be dispersed in a range of value. In absence of publication bias as shown in the present figure the studies are distributed symmetrically about the combined effect size. The pseudo 95% confidence limits illustrate the expected 95% confidence interval about the pooled fixed-effects estimate for the meta-analysis.

The funnel plot was very symmetric showing the lack of publication bias (Fig 4). The Harbord test further supports this result (p value for small-study effect = 0.765).

## Discussion

The main result of the present study, drawn from 6 RCTs including 5673 patients with SCAD followed up for a mean time of 55 months, is that in patients with SCAD an initial strategy of PCI plus OMT is not associated with an increased risk of stroke compared to OMT alone.

Although PCI is widely used for the management of patients with SCAD its role is controversial[15]. Indeed recent clinical trials(1–3) and meta-analyses [4–7] have shown that in patients with stable angina an initial PCI strategy plus medical therapy does not reduce the risk of death or MI compared to OMT alone. On the other hand from the same sources stem the evidences that PCI is associated with a better and more rapid symptoms relief. Accordingly whether a patients with stable angina should be referred to invasive management or not still represents a major question that physicians have to face in daily clinical practice. In particular among factors that may influence this choice the evaluation of intervention hazards should be taken into account. Stroke is a rare but devastating complication related to PCI[8,9]. Although its incidence, risk factors and outcome have been widely described[8,9,16–18] a direct comparison in patients with SCAD aimed to investigate if initial PCI plus OMT strategy is associated with a higher risk of stroke than OMT alone has not been performed so far.

The results of our meta-analysis, showing that the risk of stroke is not different between patients managed with PCI and those managed with OMT, are therefore noteworthy.

The strength of this findings rely on the satisfaction of all requirements for meta-analysis in term of low heterogeneity, no publication bias and sensitivity analyses.

There may be different reasons to explain our findings. First, the risk of stroke in patients undergoing PCI has been historically associated with procedural factors with an incidence during hospitalization less than 0.4%[16,17]. Guide catheter manipulation, use of anticoagulant and potent antiplatelet inhibitors may increase the risk of ischemic and hemorrhagic stroke, respectively. In a recent study(18) from the British Cardiovascular Intervention Society, including 426046 patients undergoing PCI, the incidence of 30-day ischemic and haemorrhagic stroke was 0.10% and 0.03%. Patients with SCAD were at lower risk compared to patients with ACS.

This quite low risk could also be paid by a considerable portion of patients treated with OMT alone due to the high rate of cross over during long-term follow up, realizing a sort of catch up in the risk of stroke. Indeed, the need of elective PCI in the OMT arm ranged from 9% of The Medicine, Angioplasty, or Surgery Study II study to 36.5% of the JSAP study [in the COURAGE study and Fracional Flow Reserve versus Angiography for Multivessel Evaluation 2 (FAME II) trial the rate of elective PCI for persistent angina was 32.6% and 26.5%, respectively).

Second, the risk related to PCI could be partly outfitted during follow up by the fact that patients undergoing to PCI are more likely to receive dual antiplatelet therapy compared to patients managed medically due to stent implantation routinely used in contemporary clinical practice. In fact, it is known that dual antiplatelet therapy reduce the risk of stroke as a whole compared to aspirin alone[19,20]. Notably in the present meta-analysis in order to reflect a contemporary clinical practise we included only study with a rate of stent implantation more than 50%.

### Study limitations

The results of the present study should be interpreted in light of some limitations.

This meta-analysis was based on aggregate data of randomized clinical trials that traditionally are conducted in highly-experienced centres and enrol patients with low risk clinical profile. Therefore, generalization of effect estimates should be undertaken with caution. Indeed, traditional risk factors for stroke in patients undergoing PCI such as advance age, prior stroke, atrial fibrillation and procedural complexity may be less represented in clinical trials compared to a real world setting. Given these observations whether PCI is associated or not with an increased risk of stroke as compared to OMT also in higher risk patients should be evaluated by further investigations.

Although in the present study we included only trials with > 50% of stent utilization to reflect a modern PCI practise it should be taken into account that these trials were conducted in different era in terms of type of stent, type and duration of dual antiplatelet therapy (this latter aspect related to the rate of drug eluting stent utilization).

In the present study we included more than 5000 patients; it is not excludible that a study with a larger sample size could be able to detect a significant higher risk of stroke in patients undergoing a PCI based approach compared to OMT alone. To date, however, there are no larger studies available. Nevertheless, assuming a stroke rate of 1.9% in the PCI group and 1.2% in the OMT group; i.e. 37 relative risk reduction [based on data from the Clinical Outcome Utilizing Revascularization and Aggressive Drug Evaluation (COURAGE) study] 5170 patients would be required for a study power of 80% with a type I error of 0.05. Finally, it would be of interest to evaluate also the early risk of stroke related to coronary interventions however data on peri-procedural stroke and timing of stroke were missing in most of the studies included in the present meta-analysis. Nonetheless data from recent meta-analyses [21] of 19 trials including 10944 patients randomized to coronary bypass versus PCI showed that the 30-day rate of stroke in the PCI arm was quite low (1.2% and 0.34, respectively). Yet, these findings have been confirmed also in a recent European survey showing in stable patients a rate of in hospital stroke following PCI of 0.3% [9]. Therefore, assuming a stroke rate of 0.3% in the PCI arm to detect a significant relative risk reduction of 37% in the OMT arm, with a study power of 80% and an alfa level of 0.05, 9920 patients should be recruited.

## Conclusions

In this meta-analysis of available contemporary RCTs including patients with SCAD an initial strategy based on a direct PCI is not associated with an increased risk of stroke during long-term follow up compared to an initial strategy based on OMT alone.

## Supporting Information

S1 Table PRISMA 2009 Checklist. (DOC)

S1 Text Search strategy. Combination of key words.
(DOC)

S2 Text Full-text articles excluded. Reason for exclusion.
(DOC)
