# Supplementary material for: Risk of Stroke in Patients with Stable Coronary Artery Disease Undergoing Percutaneous Coronary Intervention versus Optimal Medical Therapy: Systematic Review and Meta-Analysis of Randomized Controlled Trials
Source: PLoS One. 2016 Jul 8;11(7):e0158769. doi: 10.1371/journal.pone.0158769 (PMC4938490; doi:10.1371/journal.pone.0158769)
Supplement: S1 Text — Combination of key words. (DOC) [file pone.0158769.s002.doc]

**S1 Text. Search strategy.** Combination of key words

“stable angina and PCI”, “stable angina and stent”, “stable angina and medical therapy”, “PCI and medical therapy and stable angina”, “stent and medical therapy and stable angina”

“stable coronary artery disease and PCI”, “stable coronary artery disease and stent”, “stable coronary artery disease and medical therapy”, “PCI and medical therapy and stable coronary artery disease”, “stent and medical therapy and stable coronary artery disease”
