# Supplementary material for: Risk of Stroke in Patients with Stable Coronary Artery Disease Undergoing Percutaneous Coronary Intervention versus Optimal Medical Therapy: Systematic Review and Meta-Analysis of Randomized Controlled Trials
Source: PLoS One. 2016 Jul 8;11(7):e0158769. doi: 10.1371/journal.pone.0158769 (PMC4938490; doi:10.1371/journal.pone.0158769)
Supplement: S2 Text — Reason for exclusion. (DOC) [file pone.0158769.s003.doc]

**S2 Text. Full-text articles excluded**

**1.** Davies RF(1), Goldberg AD, Forman S, Pepine CJ, Knatterud GL, Geller N, Sopko G,

Pratt C, Deanfield J, Conti CR. Asymptomatic Cardiac Ischemia Pilot (ACIP) study two-year follow-up: outcomes of patients randomized to initial strategies of medical therapy versus

revascularization. Circulation. 1997 Apr 15;95(8):2037-43.

The study randomized 558 patients who has stable coronary artery disease with coronary anatomy suitable for revascularization to three treatment strategies: angina-guided drug therapy (n=183), angina plus ischemia-guided drug therapy (n=183), or revascularization by angioplasty or bypass surgery (n=192). This trial was excluded because compared OMT with revascularization as a whole (either PCI or coronary bypass), accordingly we were prevented from extracting data to compare OMT with PCI.

**2.** Pfisterer M. for the Trial of Invasive versus Medical therapy in Elderly patients

Investigators. Long-term outcome in elderly patients with chronic angina managed invasively

versus by optimized medical therapy: four-year follow-up of the randomized Trial

of Invasive versus Medical therapy in Elderly patients (TIME). Circulation. 2004 Sep 7;110(10):1213-8.

Patients with chronic angina were randomized to an invasive (n=153) or an optimized medical (n=148) strategy. The definition of invasive strategy included either coronary bypass or percutaneous coronary intervention preventing us from extracting data to compare OMT with PCI.

**3.** Hueb WA, Soares PR, Almeida De Oliveira S, Ariê S, Cardoso RH, Wajsbrot DB,

Cesar LA, Jatene AD, Ramires JA. Five-year follow-op of the medicine, angioplasty, or surgery study (MASS): A prospective, randomized trial of medical therapy, balloon angioplasty, or bypass surgery for single proximal left anterior descending coronary artery stenosis. Circulation. 1999 Nov 9;100(19 Suppl):II107-13.

In a single institution, 214 patients with stable angina, normal ventricular function, and severe proximal stenosis (>80%) on the left anterior descending artery were selected for the study. After random assignment, 70 patients were referred to surgical treatment, 72 to angioplasty, and 72 to

medical treatment. The reason for exclusion from the present meta-analysis was that in the PTCA arm there was no stent utilization

**4.** Bech GJ, De Bruyne B, Pijls NH, de Muinck ED, Hoorntje JC, Escaned J, Stella

PR, Boersma E, Bartunek J, Koolen JJ, Wijns W. Fractional flow reserve to determine the appropriateness of angioplasty in moderate coronary stenosis: a randomized trial. Circulation. 2001 Jun 19;103(24):2928-34.

In 325 patients for whom PTCA was planned and who did not have documented ischemia, FFR of the stenosis was measured. If FFR was >0.75, patients were randomly assigned to deferral (deferral group; n=91) or performance (performance group; n=90) of PTCA. If FFR was <0.75, PTCA was performed as planned (reference group; n=144). The study was excluded because data on stroke were not available. Besides in the OMT group (deferral group) only patients with no sign of myocardial ischemia (neither by non invasive tests or FFR) were included.
